# Application of Femtosecond Laser in Anterior Segment Surgery

**DOI:** 10.1155/2020/8263408

**Published:** 2020-04-10

**Authors:** Sang Beom Han, Yu-Chi Liu, Karim Mohamed-Noriega, Jodhbir S. Mehta

**Affiliations:** ^1^Department of Ophthalmology, Kangwon National University Hospital, Kangwon National University School of Medicine, Chuncheon, Republic of Korea; ^2^Singapore National Eye Centre, Singapore; ^3^Singapore Eye Research Institute, Singapore; ^4^Department of Ophthalmology, Yong Loo Lin School of Medicine, National University of Singapore, Singapore; ^5^Department of Ophthalmology, University Hospital, Faculty of Medicine, Autonomous University of Nuevo Leon, Monterrey, Mexico

## Abstract

Femtosecond laser (FSL) is a near-infrared laser that can create reliable and reproducible tissue cutting with minimal damage to adjacent tissue. As the laser can also create incisions with various orientations, depths, and shapes, it is expected to be a useful tool for anterior segment surgery, such as cornea, refractive, and cataract surgery. In this review, the authors will introduce the application of FSL in various anterior segment surgeries and discuss the results of studies regarding the efficacy and safety of FSL in cornea, refractive, and cataract surgery. Experimental studies regarding the potential use of FSL will also be introduced. The studies discussed in this review suggest that FSL may be a useful tool for improving the prognosis and safety of surgeries of the anterior segment.

## 1. Introduction

Femtosecond laser (FSL) is a neodymium glass (Nd:glass) laser employing ultrashort pulse durations in the femtosecond time domain (1 femtosecond = 10^−15^ sec), allowing tissue cutting with substantially reduced energy, compared with other ophthalmic laser pulses, e.g., nanosecond time domain (10^−9^ sec), argon, excimer, and neodymium yttrium aluminium garnet (Nd:YAG) lasers [1]. Such reduction in energy may result in confined tissue effect with minimal collateral damage to adjacent tissues [2].

With wavelengths in the near-infrared spectrum (1053 nm), FSL is neither absorbed by optically transparent tissues, such as cornea and lens, at low power densities, nor affected by corneal magnification [1, 2]. Infrared lasers undergo substantially reduced attenuation compared to visible wavelength lasers, and to a certain degree, FSL can transmit through haze media, such as opacified or edematous corneas.

Like the Nd:YAG laser, FSL uses a process of photodisruption, whereas argon and excimer lasers employ photocoagulation and photoablation, respectively [2]. The precisely focused FSL can increase the power density, on a targeted structure depth [3], and can cut tissue via photodisruption, which is the process of generating a plasma of free electrons and ionized molecules that rapidly expands and collapses to produce microcavitation bubbles and acoustic shock waves, resulting in incisions and separation of the target tissue [1, 4]. FSL is not only able to produce consistent and reproducible tissue incisions, but it can also allow the creation of various shapes of incisions, such as circular, decagonal, and zigzag shapes [1, 2].

The application of FSL in ophthalmic surgery was first introduced in 2001 [3]. Since then, it has been increasingly used in anterior segment surgery [3]. In corneal transplantation, FSL is applicable for customized trephination in penetrating keratoplasty (PK) and deep anterior lamellar keratoplasty (DALK) [3, 4]. It can also be used in the preparation of donor tissue for endothelial keratoplasty (EK) [3].

FSL is currently used for refractive surgery worldwide, including the creation of flaps in laser-assisted in situ keratomileusis (LASIK) and refractive lenticule extraction (ReLEx) [5]. FSL has also been increasingly used for cataract surgery, as it has been shown to improve the reliability and reproducibility for creation of corneal wound and anterior capsulotomy and reduce phacoemulsification energy for lens fragmentation [6].

In this review, we aim to provide information on the application of FSL in anterior segment surgery, including cornea, refractive, and cataract surgeries.

## 2. Femtosecond Laser in Keratoplasty

### 2.1. Femtosecond Laser-Assisted PK

**Figure 1:**
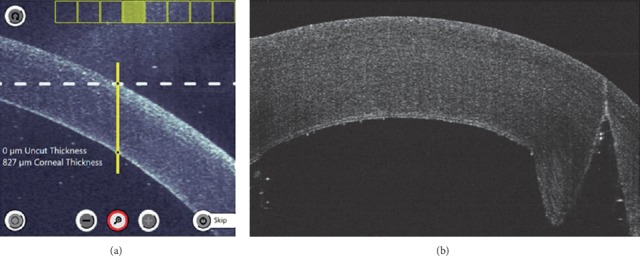
Penetrating keratoplasty with full-thickness trephination using Ziemer Z8 platform in a porcine eye model. (a) The inbuilt OCT scans in eight meridians and the depth of the laser cut can be adjusted. The yellow line indicates the pathway of laser cutting. The cutting pattern can be customized. (b) Postcutting OCT scans showing full-thickness trephination.

FSL is able to create customized trephination cuts, such as top-hat, zigzag, and mushroom configurations, to improve biomechanical wound integrity and facilitate wound healing after PK and DALK [4]. Theoretically, corneal trephination using FSL can enable the creation of more structurally stable and predictable wound configuration by providing more accurate fit with larger contact area between the donor and host (Figure 1) [7]. This may conceivably result in reduced wound distortion and enhanced wound tensile strength, decreasing surgically induced astigmatism and facilitating wound healing and visual recovery [4, 7].

Previous studies revealed that the FSL-assisted KP with the two most popular trephination patterns, the “top-hat” and “zigzag” configuration, enabled faster visual recovery with better best-corrected visual acuity (BCVA), less astigmatism, and faster suture removal than manual PK [7–12], although graft failure and rejection rates were similar to those for manual PK [8]. In comparison between the “top-hat” and “zigzag” incisions, the two patterns showed comparable visual and refractive outcomes, endothelial cell counts, and wound healing [13]. However, Chamberlain et al. [14] showed that the improvement in astigmatism with FSL-assisted PK was not significant after 6 months postoperatively, and no significant improvement in BCVA was found at any time point [14]. FSL-assisted PK with “mushroom” configuration also resulted in reduced astigmatism [15] and was suggested to be a viable option for pediatric patients, as it combined the refractive advantage of a larger anterior diameter with an immunologic advantage of smaller posterior graft [16].

### 2.2. Femtosecond Laser-Assisted DALK

With the ability to perform predictable and precise dissections at a variety of orientations and depths, while providing stable donor-host apposition, FSL is also envisaged to be a useful tool for lamellar keratoplasty procedures [17, 18]. Theoretically, FSL may be advantageous in DALK, as it enables the removal of anterior stromal lamella and formation of big bubble without difficulty [19], as well as improving wound integrity and healing [17].

**Figure 2:**
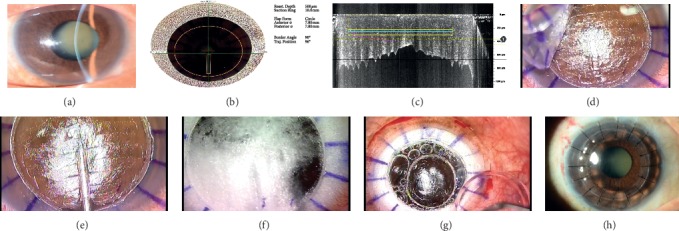
FSL-assisted DALK with Ziemer Z8 laser. (a) Preoperative anterior segment photograph of a patient with keratoconus. (b) Inbuilt intraoperative OCT for surgical planning. (c) Purple line: tunnel cut and second solid yellow line: lamellar cut. (d) Removal of anterior cap. (e) The cannula was inserted into the laser-created intrastromal tunnel for air bubble injection. (f) Injecting air for big bubble technique. (g) DM sparing and DALK graft. (h) 3 months postoperatively.

FSL can be used for the preparation of both the recipient and donor tissues [6]. In the recipient cornea, FSL first creates an anterior lamellar cut at a predetermined depth and then performs a peripheral circular trephination cut, from the lamellar interface plane to just above the corneal epithelium [3, 6, 17]. The donor tissue is prepared in a similar fashion using a corneoscleral button mounted on an artificial anterior chamber (AC), which is transferred and sutured onto the host lamellar bed using either continuous or interrupted 10-0 nylon sutures [17]. The surgical procedures of our DALK case using FSL are demonstrated in Figure 2.

A case series study demonstrated that FSL-assisted DALK was an efficient and safe procedure for visual recovery in patients with anterior corneal diseases [20]. FSL-assisted DALK with mushroom configuration enabled faster visual recovery than manual DALK [21, 22], although the final BCVA was comparable [21]. Salouti et al. [23] recently reported that FSL-assisted DALK was advantageous for reducing residual myopia and restoring corneal anatomy compared with manual DALK in patients with keratoconus, although postoperative BCVA and astigmatism were comparable.

### 2.3. Femtosecond Laser-Assisted EK

FSL can also allow for a more reliable, predictable, and precise preparation of donor and recipient tissues for EK [6]. In recipient cornea, the posterior trephination cut starts from the AC and progresses anteriorly through Descemet's membrane, and the lamellar dissection is performed on the posterior stroma [3]. The donor cut is performed in a similar fashion using a corneoscleral button mounted on an artificial AC [3].

Early results demonstrated that FSL-assisted EK showed worse visual outcome and higher endothelial cell loss than manual PK, although it had significantly reduced postoperative astigmatism [24]. The authors concluded that a modification of donor tissue insertion technique was needed to prevent endothelial cell loss [24].

Recent studies reported that FSL-assisted Descemet's membrane EK (DMEK) had a visual outcome comparable to manual DMEK, with a significantly reduced rate of graft detachment, rebubbling, and endothelial cell loss [25, 26]. Sorkin et al. [27] suggested that FSL-assisted DMEK might be a safe and effective option in patients with failed PK, resulting in substantially reduced detachment and rebubbling rates and trend towards reduced primary failure than manual DMEK.

However, FSL has a limitation that it can increase operating time and elevate the costs of tissue cutting and the surgical procedure in all kinds of keratoplasty.

## 3. Femtosecond Laser in Refractive Surgery

### 3.1. FSL-Assisted Laser In Situ Keratomileusis (FSL-LASIK)

The application of FSL in LASIK flap creation has rapidly gained popularity since its introduction in 2002 [3]. The FSL first performs lamellar dissection at a predetermined depth in the anterior stroma, creating circular vertical cuts in a posterior to anterior direction [3]. Using an instrument, such as an iris sweep, the flap interface is swept across and the flap is lifted [3].

FSL has the following advantages in flap creation over the mechanical microkeratome: (1) wide variability of flap parameters, such as flap thickness and diameter, side cut angle, hinge length, and position [28, 29]; (2) more precise and predictable flap thickness and position that result in improved flap safety [28, 29]; (3) decreased risk of flap-related complications, such as free caps, buttonholes, short flaps, and irregular cuts [28]; and (4) flaps with uniform thickness with a planar shape that is different from the mechanical flap with meniscus shape [3].

Meta-analysis studies concluded that FSL-LASIK has good visual outcome and safety comparable to LASIK with microkeratomes and may have improved predictability of flap thickness and refractive error [30, 31].

Regarding complications, diffuse lamellar keratitis is the most common but is generally mild and self-limited [30]. FSL is also associated with several unique complications. First, confluent cavitation gas bubbles during intrastromal laser treatment can result in opaque bubble layer (OBL) in the deep stromal bed that may interfere with iris registration and pupil localization [3]. Second, seepage of gas bubbles into the subepithelial space can cause flap buttonholes [32]. Although it is extremely rare, leakage of gas bubbles into the AC may hamper the centration of the laser beam [33]. Third, transient light-sensitivity syndrome is characterized by severe photophobia with good visual acuity and absence of abnormalities on ophthalmologic examination [34]. It usually occurs 2 to 6 weeks postoperatively and improves in a week with topical corticosteroids [34]. Finally, rainbow glare may be an optical side effect of light scatter from the back surface of the interface after FSL-LASIK, which could be prevented by using the improved focusing optics of higher numeric aperture [30, 35].

### 3.2. Refractive Lenticule Extraction (ReLEx)

FSL-LASIK requires both FSL for flap creation and an excimer laser for corneal stromal ablation [5]. Femtosecond lenticule extraction (FLEx) was introduced as a new method that requires only FSL [36], which was further developed into small incision lenticule extraction (SMILE) [5]. Subsequently, the overall terminology “refractive lenticule extraction (ReLEx)” was suggested to include these two procedures [5].

In FLEx, a corneal flap is created using FSL [36]. However, FLEx involves intrastromal dissection using FSL and extraction of a refractive lenticule instead of stromal ablation as in FSL-LASIK [36]. In SMILE, the flap is not created and the refractive lenticule is extracted through small peripheral corneal incision constructed by FSL [5]. Theoretically, SMILE can improve corneal biomechanical stability by avoiding corneal flap creation [36].

**Figure 3:**
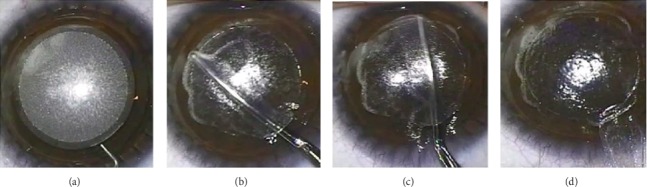
Small incision lenticule extraction (SMILE). (a) Small arcuate incision, anterior and posterior lenticule planes are cut by FSL. (b) Dissection of anterior lenticule plane, followed by (c) dissection of posterior lenticule plane. (d) Extraction of lenticule from small incision.

In SMILE, after initial docking of the eye with the interface cone and suction fixation, the FSL creates a posterior surface of the lenticule, a lenticule side cut, an anterior surface, and a small incision [37, 38]. Then, a manual dissector is inserted into the pocket through the incision, which is used to separate the lenticule along the anterior and posterior surface [37, 38]. The lenticule is then extracted through the incision using removal forceps (Figure 3) [37, 38].

Lenticule dissection and extraction is the most challenging step and can lead to complications, such as tear of anterior cap or side cut, posterior stromal damage, and partially or completely retained lenticule [39]. However, these complications are related to inexperience and may decrease with the learning curve [37, 39]. Complications, including suction loss, incisional bleeding, OBL, and inaccurate laser pulse placement, have also been reported; however, these can mostly be resolved with appropriate management [40].

Studies suggest that both FLEx and SMILE show good visual and refractive outcomes, safety, and predictability profiles [5, 41, 42]. SMILE was reported to have good efficacy and safety comparable to FSL-LASIK, and milder higher-order aberrations and spherical aberration, higher contrast sensitivity, and fewer dry eye symptoms than FSL-LASIK [43–46] A meta-analysis study indicated that SMILE may be advantageous over FSL-LASIK due to its association with a lower risk of flap-related complications, faster corneal nerve recovery, reduced corneal nerve injury, and higher-order aberrations, despite its comparable safety, efficacy, and predictability levels to FSL-LASIK [36].

## 4. Femtosecond Laser-Assisted Cataract Surgery (FLACS)

FSL has currently four applications in cataract surgery: corneal wound construction, anterior capsulotomy, lens fragmentation, and limbal relaxing incisions (LRIs) [5]. It is envisioned to improve the safety and efficacy of the cataract surgery [1, 47].

### 4.1. Corneal Wound Construction

The optimal construction of clear corneal incision (CCI) with adequate length and architecture is important for wound safety and prevention of complications associated with wound leakage, such as induced astigmatism, iris prolapse, hypotony, and endophthalmitis [1, 48]. However, manual CCI is sometimes difficult and less predictable in terms of length and shape [49] and is more prone to injuries of Descemet's membrane and gaping of the internal wound, which can result in delayed healing and increased risk of corneal decompensation [50]. FSL allows CCIs to be leak-proof and self-sealing with greater reproducibility and safety [48, 51], which may result in better wound integrity and sealability than manual CCI [1, 52].

### 4.2. Anterior Capsulotomy

Anterior capsulotomy with appropriate size and circularity is important for the positioning and performance of the intraocular lens (IOL) [53, 54]. It is also closely related to the effective lens position (ELP), which is a major determinant of IOL power calculation [55]. Inadequate size or circularity of the capsulotomy can cause tilting, decentration, or rotation of IOL and changes in ELP that can result in worse visual and refractive outcomes, with more profound effects with multifocal and toric IOLs [1, 56].

However, manual capsulorhexis is one of the most technically challenging skills in cataract surgery [57], with increased difficulty in cases with shallow AC, capsular fibrosis, weak zonule, and mature or pediatric cataracts [54]. Although creation of capsulotomy with good size, circularity, and centration has been increasingly emphasized, manual capsulorhexis is associated with substantial unpredictability and variability even for experienced surgeons [54, 58].

FSL is shown to allow for more reliable and reproducible anterior capsulotomy with enhanced centration and circularity than manual capsulorhexis [48, 54, 58–60]. FSL can substantially reduce the risk of IOL tilting or decentration, which is particularly important for multifocal IOL [59, 61]. Animal studies revealed that FSL might be associated with increased tensile strength of the capsular opening [58, 62]. FSL is also advantageous in achieving complete overlap between the anterior capsule and IOL optic, which is important for IOL centration and prevents posterior capsular opacification, compared to manual capsulorhexis [54, 60]. Dick et al. [63] reported that FLACS achieved earlier capsular bag stabilization, suggesting that it may allow for more predictable ELP, IOL power calculations, and refractive outcomes [63, 64].

FSL has another advantage. It is not influenced by the axial length, pupil size, and corneal magnification, whereas manual capsulorhexis is dependent on these factors [60].

### 4.3. Lens Fragmentation

Ultrasound energy within the AC causes oxidative stress and increases the risk of injury to the iris, capsule, and cornea [65]. FLACS involves the pretreatment of the lens using liquefaction or fragmentation to segment or soften the cataract [2]; thus, it can reduce the amount of ultrasound energy and intraocular manipulation during phacoemulsification [51, 58, 66–68]. Hence, FLACS is predicted to reduce the risk of posterior capsular rupture and corneal endothelial cell injury [49, 67].

Studies have revealed that FLACS substantially reduced phacoemulsification energy and effective phacoemulsification time (EPT) compared with conventional cataract surgery [51, 58, 66]. FLACS has also been reported to be associated with decreased corneal swelling and endothelial cell loss, which might be correlated with reduction of EPT [66, 69, 70].

### 4.4. Limbal Relaxing Incisions

With its potential ability to create precise and accurate LRIs, FSL can theoretically overcome the limitations of manual LRIs, which include technical difficulty and unpredictability, and as such, it is expected to be widely used for the correction of astigmatism [1, 2]. Chan et al. [71] suggested that arcuate keratotomy using FSL might be helpful for the management of low to moderate astigmatism after cataract surgery. Yoo et al. [72] reported that FSL-assisted arcuate keratotomy could be a safe procedure with comparable efficacy to toric IOL for reducing residual astigmatism after cataract surgery.

### 4.5. Learning Curve

The FLACS technique does require a significant learning curve, as demonstrated by Bali et al. [73], who studied the first 200 cases. Suction breaks occurred in 2.5% of cases (5 eyes), which led to an abortion of the remaining laser procedure [73]. Small anterior capsular tags were found in 10.5% of cases (21 eyes), which led to anterior radial tears in 4% (8 eyes) [73]. Posterior capsular ruptures and posterior lens dislocation occurred in 3.5% (7 eyes) and 2% (4 eyes), respectively [73]. Although these complication rates may appear even higher compared with conventional phacoemulsification, it should be noted that the report is describing the learning curve of FLACS [73]. Conventional phacoemulsification also requires a significant learning curve, as pointed out by Martin and Burton [74] the rate of vitreous loss fell from 4.0% in the first 300 cases to 0.7% in the last 300 cases, over the course of 3000 conventional phacoemulsification cases [74]. In a report of a course of the first 1500 FLACS cases, Roberts et al. [75] revealed that the incidence of anterior and posterior capsular tears significantly decreased from 7.5% (15 eyes) in the first 200 cases to 0.62% (8 eyes) in the latter 1300 cases, indicating the safety of FLACS after learning curve [75]. Other studies also reported the rate of anterior capsule tear to be in the range of 0.21%–0.43% [76, 77], suggesting that the capsular complication rate of FLACS might be lower compared with that of conventional surgery reported in the literature [78].

### 4.6. Clinical Outcome

Kránitz et al. [61] reported that the FLACS group demonstrated significantly better BCVA than the conventional surgery group, suggesting that the better BCVA showed a correlation with less IOL tilting and decentration [61] Filkorn et al. [79] showed that the FLACS group had greater predictability of IOL power calculation, with greater differences especially in the long (axial length >26.0 mm) and short (axial length <22.0 mm) eyes [79]. Miháltz et al. [80] revealed that FSL capsulotomy led to significantly reduced internal optical aberrations compared with manual capsulotomy, which might result in better optical quality [80]. Lee et al. [81] recently demonstrated that FLACS was associated with greater predictability in the astigmatic change, lower internal aberrations, and increased patient satisfaction [81].

By contrast, several studies reported that FLAC did not show any significant improvement in the refractive and visual outcomes [51, 82–84], although the reduction in EPT might validate the safety and efficacy of FLACS [84]. Roberts et al. [85] recently revealed that FLACS showed a significant reduction in posterior capsule ruptures, although it did not result in any significant differences with respect to the visual outcome, refractive error, and corneal endothelial injury.

FLACS is also suggested to be associated with decreased aqueous flare as a measure of postoperative intraocular inflammation [86, 87], which might be correlated with reduction in EPT [87]. Although FSL capsulotomy can increase the inflammatory cytokines and prostaglandin levels in AC [88, 89], the reduction of EPT energy may contribute to the reduction in postoperative AC inflammation [89]. FLACS also resulted in a significant reduction in thickness of 1.5 mm inner macular ring during the early postoperative period, suggesting that FLACS may be associated with milder postoperative inflammation and can be beneficial for patients at risk of postoperative inflammation and macular edema [1, 90].

FLACS can improve the outcomes in complicated cases, such as trauma cases with anterior capsule rupture or lens subluxation associated with Marfan syndrome [91, 92]. It is also advantageous in eyes with shallow ACs over conventional cataract surgery, offering milder AC inflammation and better visual outcome [93]. Successful FLACS after implantation of the Malyugin ring in the case of acute phacomorphic glaucoma with mature cataract and shallow AC has also been reported [94].

FSL can improve the safety of anterior capsulotomy in intumescent white cataract [64, 95]. In a study of 25 eyes with white cataract, Conrad-Hengerer et al. [95] reported that FLACS allowed for an uneventful IOL implantation in all cases, although radial tear and incomplete capsulotomy button occurred in 2 eyes (8%) and 3 eyes (12%), respectively.

In pediatric cataract, elasticity of the lens capsule renders the capsulorhexis more challenging and unpredictable, often leading to decentered, inadequate sizes of capsulotomy and even radial tears [49]. Hence, FLACS may play an important role in the improvement of the efficacy and safety of pediatric cataract surgery, especially with respect to the creation of anterior capsulotomy with good diameter and centration [49]. Dick et al. [96] reported the successful use of FLACS for both anterior and posterior capsulotomies in 4 children aged 9 months to 7 years. Fung et al. [97] recently introduced the use of the mobile FSL platform in anterior capsulotomy for pediatric cases, suggesting that FLACS can be applied in patients receiving surgery under general anesthesia [97].

### 4.7. Limitations and Complications

Corneal opacification can interfere with the absorption of the laser and cause dispersion of laser energy [1]. Hence, significant corneal opacity may hinder FLACS; however, the degree of opacity that causes significant scattering of FSL has not yet been elucidated [51]. As FSL capsulotomy requires mydriation of 7-8 mm, poor dilatation, posterior synechiae, and corectopia have been considered relative contraindication [49]. However, poor dilation can be addressed using implantation of pupil expanders, such as the Malyugin ring [49, 94].

Posterior subcapsular cataracts were also considered contraindication, due to the safety margin requirement for FLACS being at least 400 *μ*m from the posterior capsule [68]. However, Titiyal et al. [98] introduced a technique of FLACS with a hybrid pattern of cylinder and chop in which remaining outer rings acting as a protective cushion and manual hydrodissection and hydrodelineation were avoided and suggested that the techniques may be effective in cases of posterior polar cataract.

FLACS can be associated with an increased risk of capsular block syndrome, in which posterior capsule rupture and lens dislocation can occur following hydrodissection [99]. FSL lens fragmentation produces intralenticular gas, which induces nuclear volume expansion and formation of a seal between capsulotomy and the expanded nucleus. This restricts the decompression inside the lens, resulting in pressure rise on the posterior capsule and posterior capsular rupture [1, 99]. However, it can be prevented with measures, such as decompressing the AC and lens capsule before and during hydrodissection, dividing the hemispheres before hydrodissection, and performing a gentle and slow hydrodissection [1, 99].

Poor docking before laser procedure is associated with tilting of the lens, which can lead to capsular tag, incomplete capsulotomy, and incomplete lens fragmentation [49, 73]. However, these complications diminish throughout the learning curve and have also been prevented by technical developments on the interface [49, 73]. Subconjunctival hemorrhage caused by the suction ring is frequently found; however, it resolves spontaneously in 1-2 weeks [49].

Despite the potential advantages of FLACS, it has a limitation that it is not cost-effective at its current cost, because of the cost of equipment and maintenance of laser [100, 101]. FSL can also slow operating room flow for cataract surgery and increase operating time. Moreover, there are contradictory reports of the clinical comparisons between FLACS and conventional phacoemulsification surgery. A meta-analysis study concluded that FLACS was not superior to conventional phacoemulsification surgery in terms of intraoperative and postoperative complications [102]. A Cochrane systematic review including 16 randomized clinical trials that compared FLACS with conventional phacoemulsification surgery also failed to determine the superiority of FLACS [103]. Therefore, further development of FLACS would be needed to provide significant improvement in safety and efficacy and to reduce costs to keep health systems sustainable.

## 5. Femtosecond Laser in Other Anterior Segment Surgeries

### 5.1. Astigmatic Keratotomy and Arcuate Wedge Resection

FSL can lessen the burden and increase precision when performing corneal astigmatic surgery, such as arcuate keratotomy or wedge resection [104, 105]. Arcuate keratotomy performed with FSL was effective and predictable in reducing postkeratoplasty astigmatism and tended to have reduced misalignment of treatment and complications including corneal perforation [104, 105]. Ghanem and Azar [106] introduced a technique using FSL to perform corneal wedge resection, which resulted in significant improvement of astigmatism [106].

### 5.2. Intracorneal Ring Segments

Intracorneal ring segments are implanted in the midperipheral cornea stroma for correction of myopia ≤ − 3.5 diopters, milder cases of keratoconus without central scarring and post-LASIK ectasia [3, 6, 107].

FSL may be programmed to precisely create uniform channels at a specific depth for safer insertion of the segments [6]. The use of FSL for channel creation was reported to be as effective as mechanical dissection for mild to moderate cases of keratoconus and post-LASIK keratectasia [108]. Piñero et al. [109] reported that ring segment insertion using FSL had comparable visual and refractive outcomes to mechanical expander, and FSL showed more favorable aberrometric correction [109].

### 5.3. Experimental Studies regarding Potential Application of the FSL

FSL is also suggested to enable an adjustment of IOL power by increasing hydrophilicity of the target areas within the optic, creating a refractive index shaping lens within an existing IOL [110, 111]. An *in vitro* study revealed that a negative refractive index change in the laser-treated optic areas resulting from the change in hydrophilicity might be the chemical basis for an alteration of the IOL power [112]. An experimental study using a hydrophobic acrylic IOL revealed that power adjustment using FSL produced a reliable refractive change [111]. An animal study also showed that IOL power adjustment using FSL might be a precise, reliable, and biocompatible method for the correction of refractive error after cataract surgery [110].

An experimental study showed that IOL fragmentation was feasible with FSL [113]. Anisimova et al. [114] introduced a case in which a one-piece acrylic hydrophobic IOL was successfully transected using FSL with low energy parameters for explantation via a small corneal incision. Bala et al. [115] also reported two cases in which FSL was used to transect hydrophilic acrylic IOL.

*In vitro* studies showed that the creation of gliding planes using FSL inside the crystalline lens tissue can enhance the deformation ability of the lens, suggesting that it can be a possible option for the treatment of presbyopia [116–118].

**Figure 4:**
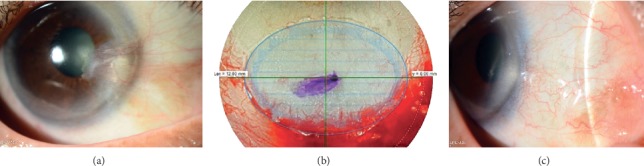
Pterygium excision and FSL-assisted conjunctival autograft preparation. (a) Preoperative anterior segment photograph. (b) Laser handpiece docking at superior bulbar conjunctiva to harvest the conjunctival autograft. The depth of the lamellar cut, and the size of autograph, can be adjusted intraoperatively. (c) At postoperative 6 months. No recurrence with good cosmetic outcome.

**Figure 5:**
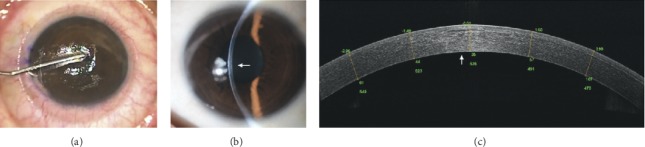
Implantation of the inlay derived from SMILE lenticule for the treatment of presbyopia. (a) Dissection of intrastromal pocket for inlay implantation. (b) At postoperative 6 months. The inlay was in the central cornea without eliciting stromal haze or inflammatory response. (c) Postoperative 6 months OCT showing the position of implanted inlay.

FSL can also enable automated, quick, and reliable preparation of an ultrathin conjunctival autografting, which might be helpful for further standardization of a surgical procedure for conjunctival reconstruction [119]. Our studies have shown the efficacy of FSL in the preparation of an ultrathin conjunctival autografting after excision of pterygium or conjunctival melanosis (Figure 4) [119–123]. Our study using a primate model suggested that biological corneal inlays derived from lenticules extracted from SMILE might be a viable option for the management of presbyopia (Figure 5) [124]. Potential application of FSL for tissue preparation for stromal keratophakia has also been introduced [125].

## 6. Conclusion

FSL is capable of precise, accurate, and predictable tissue cutting with minimal collateral tissue damage and can create customized incisions with various shapes [17, 18]. Therefore, the laser is expected to be helpful for surgeries of anterior segment tissues, including cornea and lens. So far, many studies have indicated that FSL can be a useful tool for the improvement of efficacy and safety of keratoplasty, refractive surgery, and cataract surgery. Moreover, experimental studies suggested the novel application of FSL, such as IOL power adjustment, IOL fragmentation, presbyopic correction, and pterygium surgery.

With technological development, FSL is envisaged to be an even more useful tool for various anterior segment surgeries, which will enable better prognosis and safety of these surgeries. However, the results must be validated through well-conducted clinical trials.
